# Molecular Characterization of an Intact p53 Pathway Subtype in High-Grade Serous Ovarian Cancer

**DOI:** 10.1371/journal.pone.0114491

**Published:** 2014-12-02

**Authors:** Takahide Hayano, Yuki Yokota, Kazuyoshi Hosomichi, Hirofumi Nakaoka, Kosuke Yoshihara, Sosuke Adachi, Katsunori Kashima, Hitoshi Tsuda, Takuya Moriya, Kenichi Tanaka, Takayuki Enomoto, Ituro Inoue

**Affiliations:** 1 Division of Human Genetics, National Institute of Genetics, Mishima, Japan; 2 Department of Obstetrics and Gynecology, Niigata University Graduate School of Medical and Dental Sciences, Niigata, Japan; 3 Department of Basic Pathology, National Defense Medical College, Tokorozawa, Japan; 4 Department of Pathology, Kawasaki Medical School, Kurashiki, Japan; 5 Niigata Medical Center Hospital, Niigata, Japan; University of Zurich, Swiss Institute of Bioinformatics, Switzerland

## Abstract

High-grade serous ovarian cancer (HGSOC) is the most aggressive histological type of epithelial ovarian cancer, which is characterized by a high frequency of somatic *TP53* mutations. We performed exome analyses of tumors and matched normal tissues of 34 Japanese patients with HGSOC and observed a substantial number of patients without *TP53* mutation (24%, 8/34). Combined with the results of copy number variation analyses, we subdivided the 34 patients with HGSOC into subtypes designated ST1 and ST2. ST1 showed intact p53 pathway and was characterized by fewer somatic mutations and copy number alterations. In contrast, the p53 pathway was impaired in ST2, which is characterized by abundant somatic mutations and copy number alterations. Gene expression profiles combined with analyses using the Gene Ontology resource indicate the involvement of specific biological processes (mitosis and DNA helicase) that are relevant to genomic stability and cancer etiology. In particular we demonstrate the presence of a novel subtype of patients with HGSOC that is characterized by an intact p53 pathway, with limited genomic alterations and specific gene expression profiles.

## Introduction

The age adjusted rates of ovarian and other uterine adnexa cancers in 2002 were 10.6 per 100,000, and 5.2 per 100,000 person-years in USA and Japan, respectively [1]. Epithelial ovarian cancer is a heterogenous entity comprising multiple histological types such as high-grade serous, low-grade serous, clear cell, endometrioid, and mucinous cancers [2], [3]. Ovarian cancers are divided into Type I and Type II tumors [2], [4]; Type I tumors include low-grade serous, low-grade endometrioid, clear-cell, and mucinous carcinomas. These tumors poorly respond to platinum-based therapy, harbor a high frequency of mutations in genes that encode components of the RAS signaling pathway, and are relatively stable in genomic structure. Type II tumors include high-grade serous and high-grade endometrioid carcinomas and are highly aggressive. A large-scale study of high-grade serous ovarian cancer (HGSOC) by The Cancer Genome Atlas (TCGA) group characterized HGSOC as *TP53*-mutation enriched (96%) with aberrations of genome-wide somatic gene copy numbers. This study identified commonly altered pathways such as RB1, PI3K/RAS, NOTCH, homologous recombination, and FOXM1 pathways [5]. The mutation status of *TP53* is associated with stages, gene expression patterns, and the survival of patients with serous ovarian cancer [6].

We attempted to establish a risk classification system for serous ovarian cancer using gene expression profiles acquired using microarray data [7], [8]. We identified 88 genes related to progression-free survival in 110 Japanese patients with advanced-stage serous ovarian cancer [7], as well as 126 genes related to overall survival in 260 Japanese patients with advanced-stage HGSOC [8]. To provide a better understanding of the molecular mechanisms involved in the pathogenesis of these cancers and to develop a risk classification system, we conducted profiling of the somatic mutations present in these tumors.

We compiled genomic information for patients with HGSOC using exome sequencing and copy number variation (CNV) analyses. According to the profiles of somatic single nucleotide variants (SNVs), small insertions and deletions (indels), and CNVs, we classified HGSOC into subtypes designated ST1 and ST2 that are characterized by intact or impaired p53 signaling pathways, respectively. We further characterized the two subtypes by comparing their gene expression profiles. Gene ontology (GO) analysis showed that differentially expressed genes were significantly enriched in the mitosis and DNA helicase GO groups that may be involved in genomic instability and tumorigenesis of HGSOC. These findings provide new insights into the molecular characteristics and novel biological processes that contribute to the pathogenesis of HGSOC, particularly in patients with an intact p53 pathway.

## Materials and Methods

### Ethics statement

The ethics committees of Niigata University (IRB No. 239, 428, and 455) and National Institute of Genetics (IRB No. 23-11) approved the study protocols, and each participant provided written informed consent for the collection of samples and subsequent analyses.

### Clinical samples

Fresh-frozen samples were obtained from primary tumor tissues before administration of chemotherapy. Two pathologists assessed the histological characteristics of formalin-fixed and paraffin-embedded hematoxylin and eosin sections. Because definite histological characterization was a critical component of the study, a central pathological review was conducted by two independent gynecologic pathologists (HT and TM) with no knowledge of the patients’ clinical status. Histological types and degree of histological differentiation were determined according to the WHO classification of ovarian tumors and Silverberg classification, respectively [8]. Clinical data (pT- and FIGO-stage) are shown in Table S1. We used peripheral blood as the matched normal tissue.

### Exome sequencing

Genomic DNA was isolated from tumor tissues using a phenol-chloroform method and from peripheral blood using the QIAamp DNA Blood Maxi Kit (QIAGEN) [8]. Genomic DNA was hybridized with SureSelect Human All Exon Kits (Agilent) to prepare sequencing libraries, and the libraries were sequenced using the Illumina HiSeq 2000 (Illumina) with 90 or 100 base-paired end modules. Sequence reads were aligned to a reference genome (UCSC hg19) using BWA [9] and SAMtools [10]. Picard (http://picard.sourceforge.net) was used for removing duplicate reads. Local realignment of reads around known indels and recalibration of base quality were performed using GATK [11]. The heuristic somatic mutation caller, VarScan 2 [12], was used for somatic mutation calling. Threshold criteria for detecting somatic mutations were as follows: normal variant frequency of 0% and Fisher’s exact test p value of <0.00001. Functional information of somatic mutations was annotated using ANNOVAR [13] and Oncotator (http://www.broadinstitute.org/oncotator/).

### Prediction of functional impacts of missense single nucleotide variants

Functional effects of the identified somatic missense mutations were evaluated using MutationAssessor 2 [14], which predicts the effect of amino acid substitutions according to a pattern of evolutionary conservation based on multiple sequence alignments of a protein family. Missense mutations with a functional impact score (FIS) of >2.0 were defined as deleterious.

### Detection of cancer driver genes

To detect possible cancer driver genes based on the identified somatic mutations, we used OncodriveFM [15], which evaluates the accumulation of mutations with high functional impact within a gene, assuming that cancer driver genes are highly mutated and exert substantial functional impacts. However, the consequences of mutations in passenger genes are mostly benign. OncodriveFM derives FIS from the MutationAssessor 2 to assess whether mutated genes are drivers or passengers.

### Analyses of CNV and tumor purity

Single nucleotide polymorphism (SNP) array experiments using Genome-Wide Human SNP 6.0 (Affymetrix) were previously conducted for 30 of 34 HGSOC samples [8], [16]. Because of the technical difficulties and limited DNA amounts, we could not obtain SNP array data for remaining four samples. Affymetrix CEL files from SNP array experiments using 30 samples were processed using the CNV detection software package PennCNV [17]. CNVs were called using a hidden Markov model according to calculations of the log R ratio and B-allele frequency values. The CNV frequency between tumor and normal samples was evaluated for each SNP using Fisher’s exact test in the ParseCNV algorithm [18]. Threshold criteria for recurring CNV regions (CNVRs) were as follows: Fisher’s exact test p value of <0.0005 and no overlap with structural variations in samples from healthy subjects [19]. In addition, the CEL files were used to estimate tumor purity. We used the ASCAT (Allele-Specific Copy number Analysis of Tumors) algorithm [20] in the NEXUS copy number software version 6.0 (BioDiscovery) [21] to estimate the extent of contaminations of normal cells in tumor samples. The MIAME-compliant SNP array data were deposited to the Gene Expression Omnibus data repository (accession number GSE61237).

### Microarray experiments and data processing

Extraction of RNA, Cy3 labeling, microarray hybridization, signal scanning, and feature extraction were performed in previous studies [7], [8]. Data normalization was performed using the GeneSpringGX11 (Agilent) setting of raw signal threshold of 1.0 and normalization to the 75^th^ percentile.

### Gene expression analysis

The significance of differences in gene expression between the two subtypes was evaluated using the *t*-test. After the evaluation, multiple testing was corrected by the false discovery rate (FDR) using the Benjamini-Hochberg procedure [FDR (BH)]. We set FDR (BH) to <0.1 as the significant threshold. These analyses were performed using the ComparativeMarkerSelection module of GenePattern [22].

### GO analysis

GO analysis was performed using the Functional Annotation Clustering tool included in the DAVID bioinformatics resource [23]. This tool assesses the similarity of annotation terms using kappa statistics and forces groups to share similar annotation profiles using a fuzzy heuristic multiple-linkage partition [24]. Settings were as follows: eight annotation categories (OMIM_Disease, COG_Ontology, SP_PIR_Keywords, GOterm_BP_FAT, GOterm_MF_FAT, BBID, BioCarta, and KEGG_Pathway), similarity term overlap of ≧3, kappa statistic threshold of 1, group membership of ≧3, and the fuzzy multiple-linkage partition threshold of 1, respectively. Enrichment scores were calculated using the geometric mean of the modified Fisher’s exact test p values (−log scale) for gene enrichment of each GO term in each GO group and an enrichment score of >1.3 is considered significant [23].

### Data visualization

Somatic mutation data were displayed using Gitools (version 1.8.4) [25]. Copy number data were displayed using Integrative Genomics Viewer (IGV, version 2.3.25) [26]. Bee swarm and box plots were created using the beeswarm package in the CRAN repository (http://cran.r-project.org/). Heat map views of gene expression data were displayed using HeatMapViewer module in GenePattern [22].

## Results

### Genomic alteration profiling

**Figure 1:**
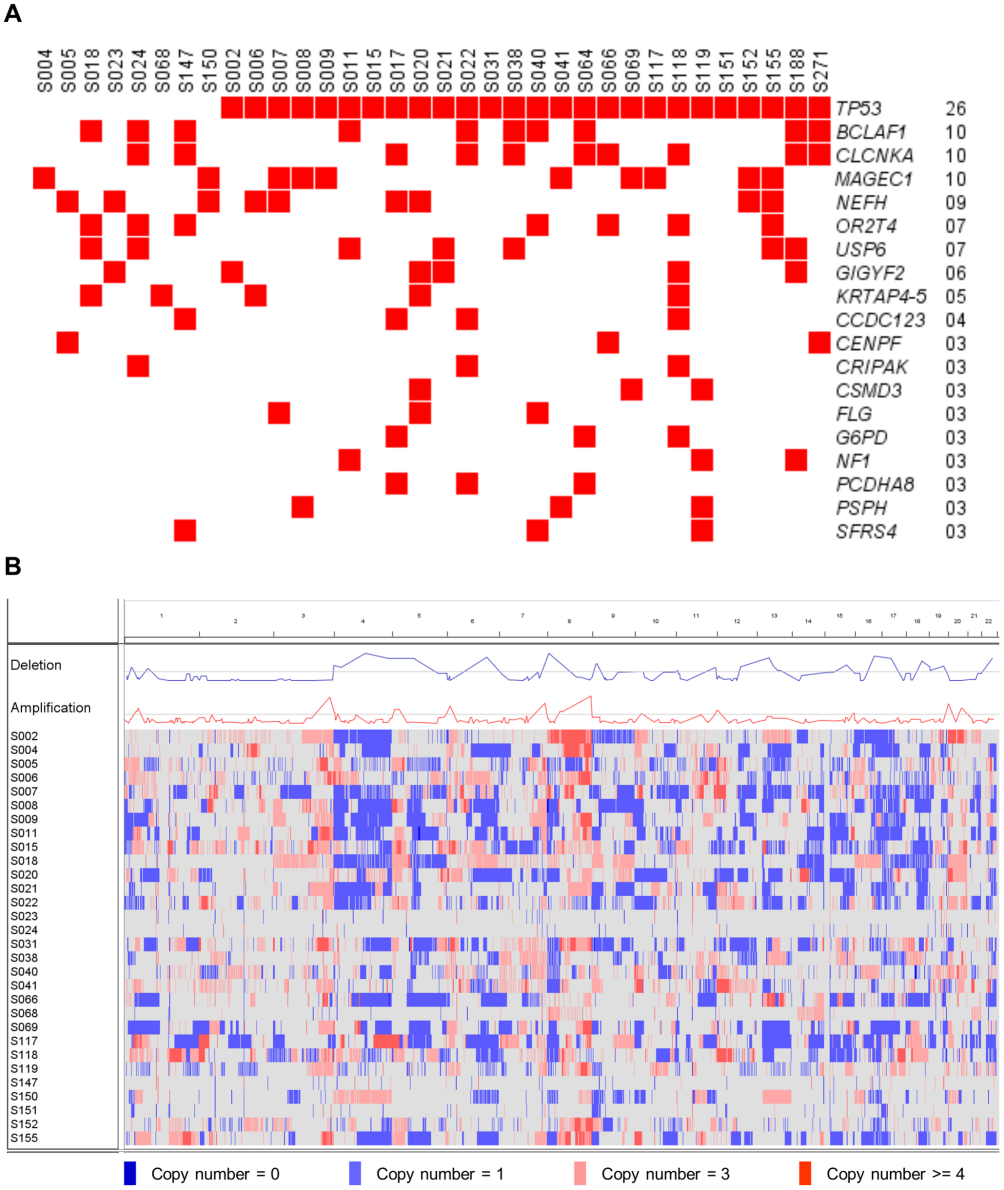
The landscape of genomic alterations in patients with HGSOC. (A) Somatic mutational landscape of 34 patients with HGSOC. Somatic mutations identified in more than or equal to three patients are displayed. Patients with mutations of the same gene are shown in red. (B) Copy number alteration landscape of 30 patients with HGSOC. Copy number (CN) alterations are indicated as follows: CN = 0, dark blue; CN = 1, light blue; CN = 3, pink; and CN ≧4, red). Blue line in the Deletion track and red line in the Amplification track show copy number alteration frequency. Gray lines in the Deletion and Amplification tracks show the −log-transformed Fisher’s exact test p values of 0.0005.

The somatic mutations identified in samples acquired from 34 Japanese patients with HGSOC were catalogued according to the analysis of exome sequencing data. The average read depth was 91× and 84× for tumor and normal samples, respectively. Coverage of ≧10× was achieved for 89% and 88% of coding bases of tumor and normal samples, respectively (Table S2). We identified 1,399 somatic nonsynonymous (missense and nonsense) and splice site mutations (41 mutations per sample) using VarScan 2 [12] with the predefined criteria described in the Materials and Methods section. Of these somatic variants, 158 were randomly selected and subjected to Sanger sequencing, and 143 variants were successfully validated (143/158, 91%). All *TP53* somatic nonsynonymous and splice site mutations were called and validated using VarScan 2 and Sanger sequencing, respectively. For nine patients with no *TP53* somatic nonsynonymous and splice site mutations, we further performed Sanger sequencing for all of the ten *TP53* coding exons because false negative might be expected due to existing low depth reads. We detected a frame-shift deletion on exon 3 for S022 (Table S3). Somatic SNVs and indels were annotated to 1,405 in 1,159 genes. *TP53* was the most frequently mutated (76%, 26/34) (Figure 1A), however the mutation frequency was lower than previous reports [5], [27]. There were 24 distinct and diverse *TP53* mutations (Table S4). Two patients (S066 and S271) shared the same missense variant (R273H) and the other two patients (S009 and S017) shared the same nonsense variant (R196*). Of the remaining 22 *TP53* variants, five were frame-shift deletions (A86fs for S020, P27fs for S022, F113fs for S119, S241fs for S006, and E286fs for S118), one was a nonsense variant (Q52* for S015), two were splice site variants (Y126splice for S188 and S261splice for S008), and the remaining 14 were missense (Table S4). FIS for the 15 *TP53* missense variants was >2.0 according to MutationAssessor 2 [14] analysis and were designated as deleterious (Table S4).

The second most frequently mutated genes were *BCLAF1*, *CLCNKA*, and *MAGEC1* (29%, 10/34 for each gene). According to FIS determined using MutationAssessor 2, all mutations except K911fs of *BCLAF1* were assessed as benign and were considered passenger mutations. Ninety-two percent (1,063/1,159) of the genes were mutated in one patient. To further explore candidate cancer driver genes mutated in at least two patients, OncodriveFM was applied as described in Materials and Methods section. Only *TP53* was detected as a cancer driver gene with high accumulation of deleterious mutations in our HGSOC samples (data not shown).

CNV profiling for 30 of the 34 HGSOC samples is shown in Figure 1B and File S1. The genome-wide copy numbers of 30 HGSOC samples were altered. ParseCNV identified nine repeatedly deleted CNVRs (1p36.11, 4q24, 5q13.1, 5q13.2, 6q22.33-23.1, 15q24.2-24.3, 17q12, 18q21.31, and 22q12.3) and four amplified CNVRs (1p34.1-33, 3q27.2, 6p24.2, and 10p12.31-12.2) with identified genes, respectively (Tables S5 and S6).

### Exclusion of p53 pathway-impaired patients from nonmutated TP53 HGSOC

**Figure 2:**
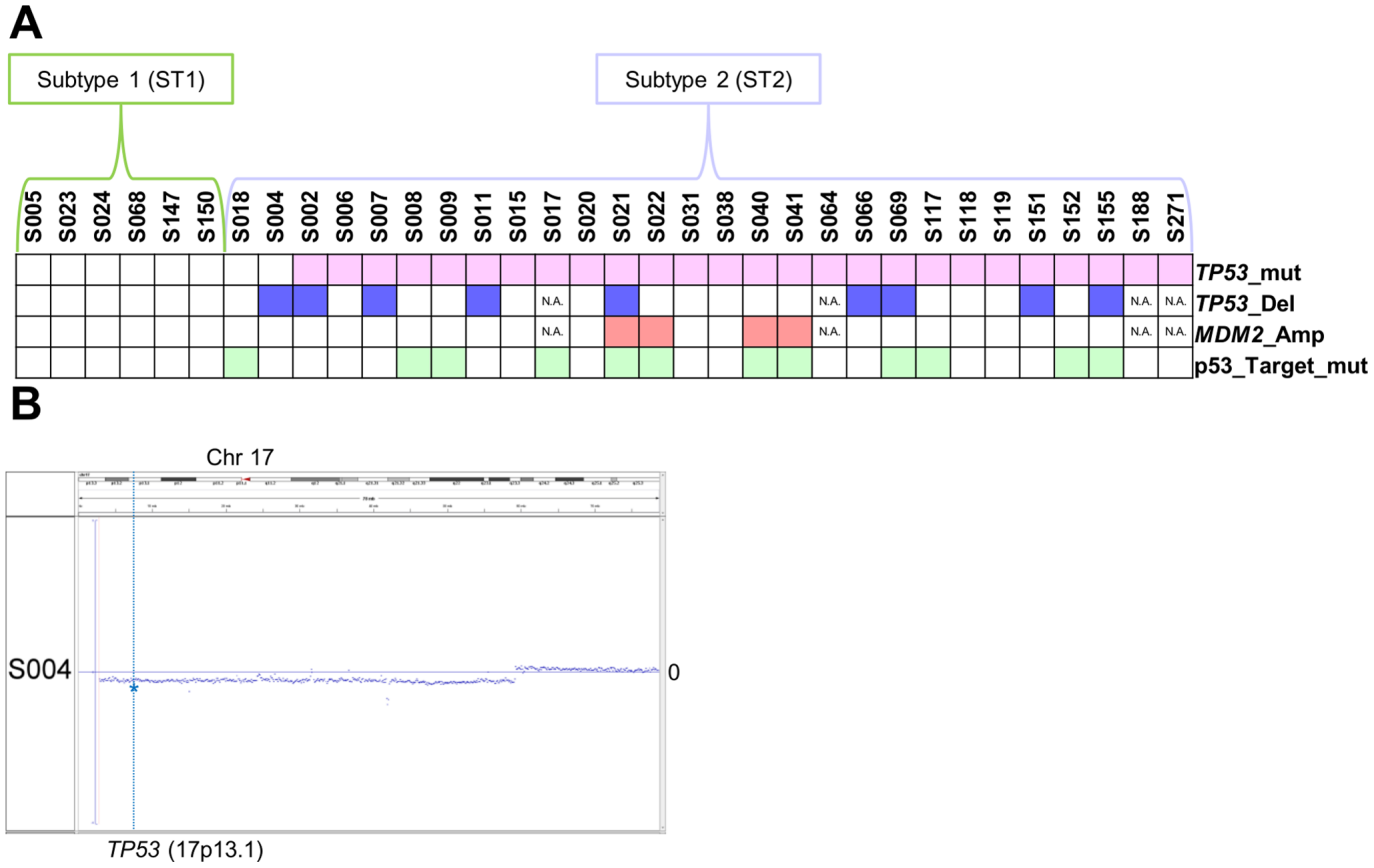
Summary of mutations for *TP53* and p53 pathway genes. (A) Summary of patients with *TP53* mutations are shown in pink in the *TP53*_mut track. *TP53* heterozygous copy number deletions are shown in blue in *TP53*_Del track. *MDM2* copy number amplification is shown in red in the *MDM2*_amp track. Mutations in genes that are direct targets of p53 are shown in green in the p53_Target_mut track. (B) Dot plot of log R ratio (LRR) of Chr17 for sample S004. Blue dots indicate LRR values. The position of line of LRR = 0 is indicated as 0 on the right of each graph. *TP53* (17p13.1) is indicated by the blue asterisk on the vertical line.

The *TP53* mutation frequency was significantly lower in our samples compared with those reported in previous studies as follows: 26/34 vs. 301/316; Fisher’s exact test p value of 0.0060 [5] and 26/34 vs. 118/126; Fisher’s exact test p value of 0.0069 [27]. Among the eight samples with nonmutated *TP53* (Figure 2A), CNV analysis showed heterozygous copy number loss of *TP53* for sample S004 (Figure 2B). MDM2 is an E3 ubiquitin protein ligase that targets p53 for proteasomal degradation and is considered a negative effector of p53 [28]. There is an association between amplification of *MDM2* and loss of p53 function in certain tumors [27]. For the eight samples with intact *TP53*, no *MDM2* copy number amplification was observed (Figure 2A). To further investigate whether an alternative mechanism accounts for p53 dysfunction, we evaluated a list of direct p53 target genes (Table S7) obtained from the Pathway Interaction Database (PID) [29]. We identified an *IRF5* (Interferon Regulatory Factor 5) splice site mutation (W181splice) of sample S018. Overall, we identified six p53 pathway intact patients from the eight patients with HGSOC with nonmutated *TP53* (Figure 2A). We assigned six patients to ST1 and the remaining to ST2.

### Genomic alterations in ST1 and ST2

We did not detect mutations in genes specific for low-grade serous type, such as *BRAF*, *CTNNB1*, *KRAS*, and *PIK3CA* [27], among the 1,159 genes mutated in the 34 HGSOC samples (data not shown).

**Figure 3:**
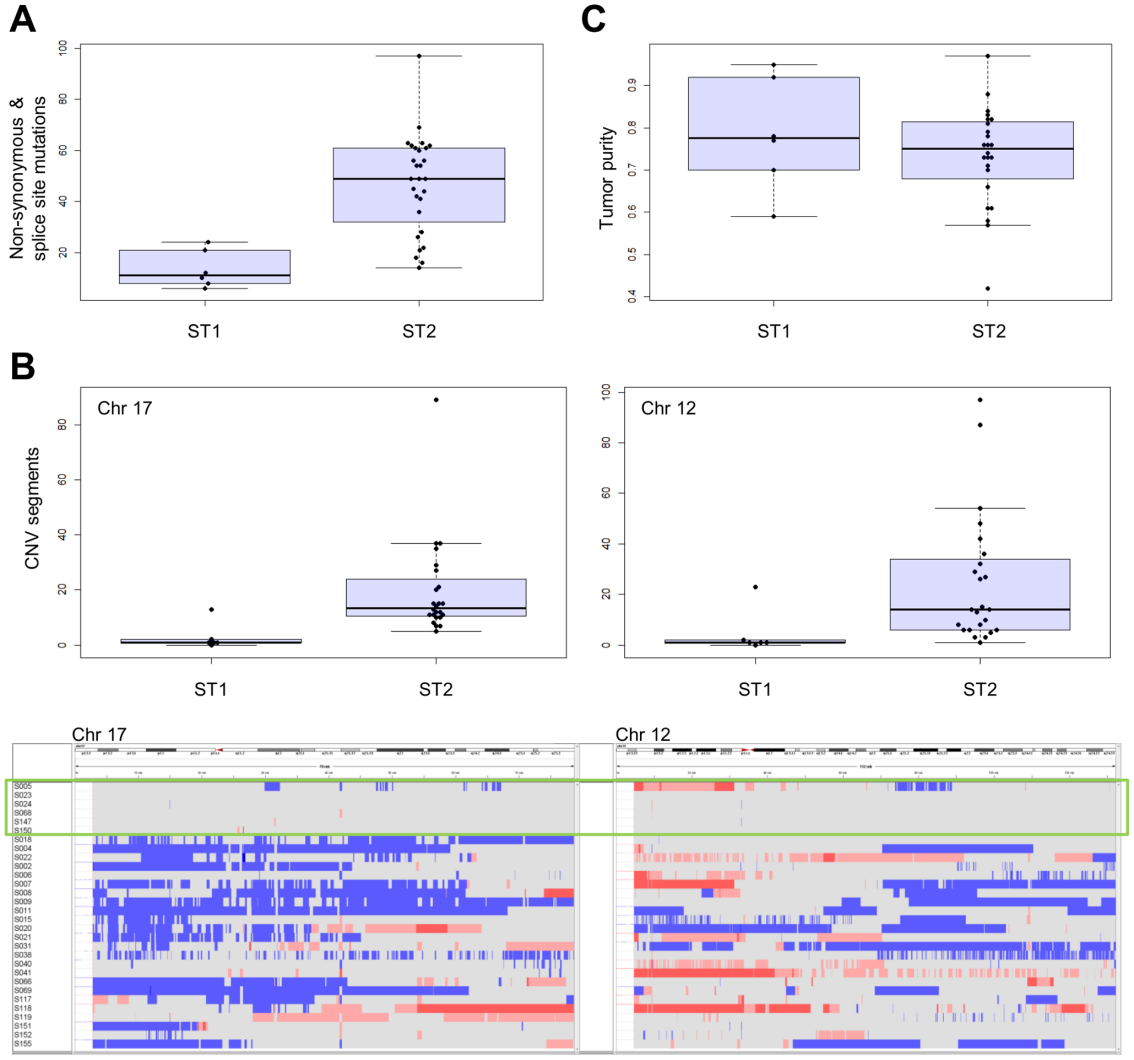
Analysis of genomic alterations in ST1 and ST2. (A) Comparison of the number of somatic nonsynonymous and splice site mutations. (B) Comparison of the number of CNV segments (upper panel) and CNV profiles (bottom panel) on chromosomes 17 and 12 between ST1 and ST2. Copy number alterations are as follows: CN = 0, dark blue; CN = 1, light blue; CN = 3, pink; and CN ≧4, red). ST1 is enclosed by the green rectangle. (C) Tumor purities of ST1 and ST2.

To characterize differences in genomic alterations between ST1 and ST2, we compared the numbers of somatic nonsynonymous and splice site mutations and found the number of somatic ST1 mutations was significantly lower compared with ST2 (Wilcoxon rank sum test p value of 0.00070) (Figure 3A).

In addition, we compared ST1 and ST2 with respect to the numbers of CNV segments identified by PennCNV [17] in each autosomal chromosome (Table S8). The results of the Wilcoxon rank sum test and multiple test correction for 22 autosomal chromosomes according to false discovery rate (FDR) [30] showed significantly fewer CNV segments on chromosomes 17 and 12 (FDR q value of 0.040 and 0.047, respectively) for ST1 (Figure 3B). These results indicate that ST1 maintained the normal karyotype and ST2 harbored genome-wide copy number alterations particularly enriched in chromosomes 17 and 12 (Figure 3B).

To exclude the possibility that the low number of mutations and few CNV segments of ST1 were because of a high degree of contamination with normal cells, tumor purity was evaluated as described in Materials and Methods section. The average tumor purities were 79% and 73% for ST1 and ST2, respectively, and there was no significant difference in tumor purity between subtypes (Wilcoxon rank sum test p value of 0.48) (Figure 3C and Table S9).

### Gene expression analysis to functionally characterize ST1 and ST2

**Figure 4:**
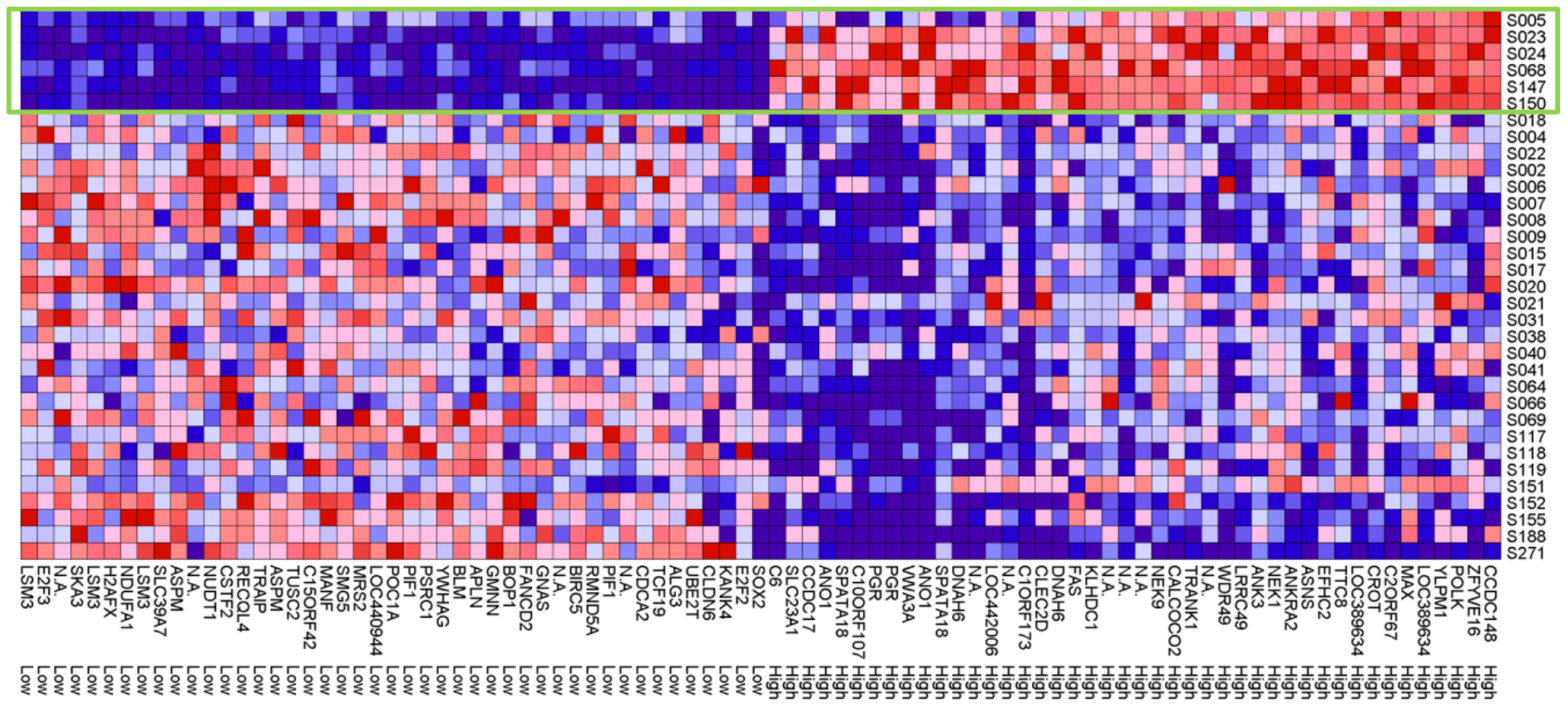
Analysis of gene expression. Seventy genes (89 probes) showing differences at FDR (BH) of <0.1 are displayed. ST1 is enclosed by the green rectangle. High and Low indicate expression levels of ST1 compared with ST2.

The gene expression profiles of ST1 and ST2 were determined using an mRNA microarray [7], [8]. Eighty-nine probes representing 70 genes revealed differences in expression levels between ST1 and ST2 at an FDR (BH) of <0.1 (Tables S10 and S11). The expression levels of 33 and 37 genes were higher (Table S10) and lower (Table S11), respectively, for ST1 compared with that for ST2. The 70 genes showed relatively homogenous and heterogenous expressions in ST1 and ST2, respectively (Figure 4).

**Figure 5:**
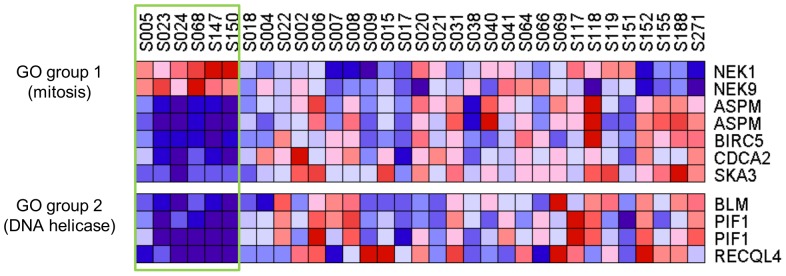
Gene ontology analysis of differentially expressed genes. Heat-map view of gene ontology (GO) groups. Two GO groups (mitosis and DNA helicase) with significant gene enrichment are indicated as GO groups 1 and 2, respectively. ST1 is enclosed by the green rectangle.

**Figure 6:**
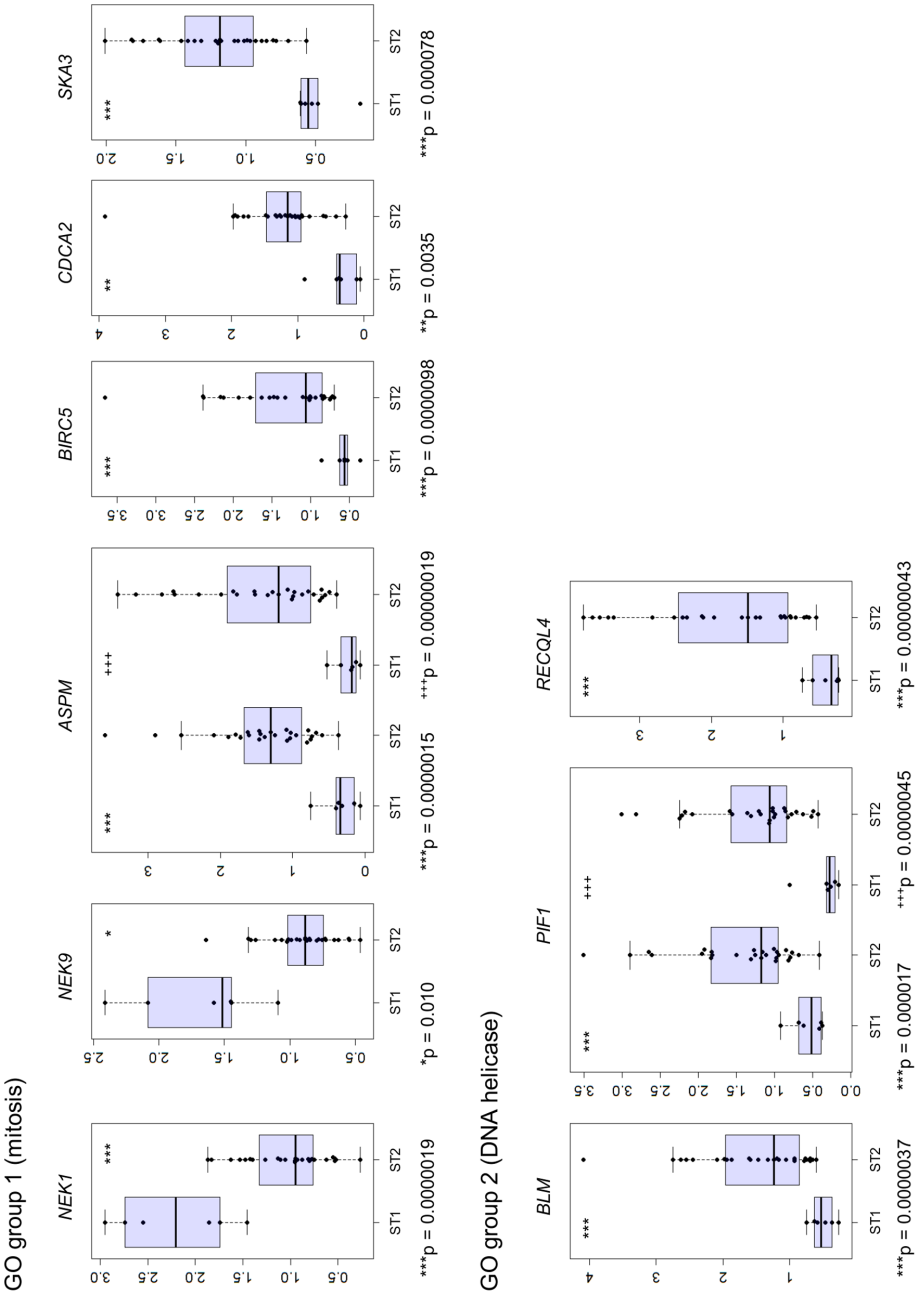
Analysis of the expression of mitotic and DNA helicase genes. Bee swarm and box plots display the gene expression pattern of ST1 and ST2 patients. Y axis indicates normalized gene expression signals processed by GeneSpring. Asterisks (*) or plus signs (+) indicate *t*-test p values as follows: *p<0.05, **p<0.01, and *** ^(+++)^p<0.001.

To evaluate the biological and functional consequences of the expression of these 70 genes, GO analysis was applied using DAVID. Thirty-five genes were classified into 18 GO groups sharing similar GO terms (Table S12). Two of the 18 GO groups (mitosis and DNA helicase) showed significant enrichment of genes (Enrichment score of >1.3) (Figure 5 and Table S12). *NEK1* and *NEK9* in the mitosis group were upregulated and *ASPM*, *BIRC5*, *CDCA2*, and *SKA3* were downregulated in ST1 compared with that in ST2. *BLM*, *PIF1*, and *RECQL4*, which encode DNA helicases, were expressed at relatively low levels in ST1. Differences in expression of these mitosis and DNA helicase genes were evaluated using the Kolmogorov-Smirnov test, F test, and *t*-test with R version 3.0.2 (Figure 6 and Table S13).

## Discussion

The analyses of somatic mutations of HGSOC showed enrichment of *TP53* mutations (Figure 1A). The CNV analysis revealed an altered profile of the genome-wide copy number (Figure 1B). These findings are consistent with those of a previous study [5]. However, we detected a significant difference in the frequency of *TP53* mutations compared with that reported in previous reports [5], [27]. Specifically, eight HGSOC samples did not harbor *TP53* mutations, and mutation of a p53 target gene *IRF5* was identified in one sample. Further, one had *TP53* copy number deletion. Taken together, we assigned six HGSOC samples as ST1 and the remaining 28 samples as ST2.

All of the patients with HGSOC in this study were Japanese while the patients in the previous studies [5], [27] were mainly come from European-descendent populations. The discrepancy of *TP53* mutation frequencies may come from population differences as observed in the case of epidermal growth factor receptor (*EGFR*) mutations for non-small-cell lung cancers [31], [32]. *EGFR* mutation rates were as follows: 11% and 32% in West-European and East-Asian patients, respectively [31], and 2% and 26% of patients in USA and Japan, respectively [32]. The low numbers of patients in the current study compared to the TCGA data set [5] may not enough to provide solid conclusion of the *TP53* mutation frequency. Evidently, much larger scale study including Japanese and other Asian patients with HGSOC are needed. The other possibility is the existence of small fraction of *TP53* mutated tumor cells because of tumor heterogeneity in the *TP53* nonmutated patients. It is widely accepted that somatic driver mutations such as mutations of *TP53* occur at an early event of cancer then relatively high frequency of the mutation should be observed. In the current study, we indeed observed at least 20% of tumor variant frequencies for *TP53*. Therefore, we presumably did not overlook driver mutations of *TP53* by the exome sequencing (Figure 1).

The low numbers of somatic mutations and CNV segments observed in ST1 likely reflect a functionally intact p53 pathway. ST2 was enriched for *TP53* mutations, and genome-wide copy number profiles were similar to those of Type II tumors. In contrast, *TP53* was nonmutated in ST1 and exhibited a normal karyotype similar to that of Type I tumors as proposed in a previous review [2]. However, we did not detect mutations in genes encoding components of the RAS signaling pathway in ST1 (data not shown). In the largest dataset from TCGA [5], 15 of 316 samples from patients with HGSOC harbored nonmutated *TP53*. When we searched for *TP53* deletions, *MDM2* amplification, or p53 target-gene mutations in the 15 samples, only one (TCGA-25-1328) was classified as ST1 (Figure S1). Hierarchical clustering using 45 overlapping genes among the 70 differentially expressed genes assigned TCGA-25-1328 to ST1 (Figure S1, bottom). These results imply that ST1 is a novel HGSOC subtype based on mutation and CNV profiles.

To further characterize the functional characteristics of ST1 and ST2, we compared their gene expression profiles (Figure 4). Using a significance threshold [FDR (BH) <0.1], we identified 70 genes that were homogeneously expressed in the ST1 microarray and heterogeneously expressed in the ST2 microarray (Figure 4). The heterogenous gene expression of ST2 may indicate diversification of molecular subtypes as secondary events as proposed in the review cited above [2], and homogeneous gene expression of ST1 may reflect an early event of oncogenesis before chromatin instability occurs.

GO analysis identified 18 GO groups that share highly similar biological terms, and two groups were significantly enriched for genes involved in mitosis and those that encode DNA helicases (Figures 5 and 6). Defects in mitosis lead to abnormal chromosome numbers that is associated with oncogenesis [33]. Two mitotic genes encoding the kinases NEK1 and NEK9 were highly expressed in ST1, and upregulation of these kinases is associated with genomic stability and tumorigenesis [34]–[37]. Moreover, other mitotic genes (*ASPM*, *BIRC5*, *CDCA2*, and *SKA3*) were highly expressed in ST2, and aberrant activation of the expression of these genes is associated with oncogenesis [5], [38]–[42].

DNA helicases maintain genome stability through DNA repair, recombination, and replication. The DNA helicases, BML and RECQL4, are inactivated in cancer prone genetic disorders such as Bloom and Rothmund-Thomson syndromes [43], [44]. Upregulation of DNA helicase expression commonly occurs in several cancers (e.g., hematopoietic, prostatic, and hepatocellular) [43]–[48]. Elevated expression of the DNA helicase genes *BLM*, *PIF1*, and *RECQL4* which is generally observed in cancers may explain a recovery function from chromatin instability in ST2. In contrast, decreased expression of genes encoding DNA helicases that characterized ST1 indicates that chromatin instability does not occur in ST1. Further investigations are required to clarify the relationship between expression of these genes and the pathogenesis of HGSOC.

We did not detect differences in overall or progression-free survival of patients classified as either ST1 or ST2 (Figure S2). All samples were diagnosed as high-grade cancer by pathologists, and the samples classified as ST1 were retrospectively examined; however, they lacked unique pathological features. ST1 was characterized by an intact p53 pathway; however, there were no differences in patients’ pathological findings or clinical consequences. These findings suggest the presence of unidentified biological processes involved in the ST1 phenotype, indicating that a more effective therapy must be developed for these patients.

In summary, we describe the identification of a novel intact p53 pathway subtype in Japanese patients with HGSOC. Our findings promise to enhance our understanding of the molecular mechanisms of oncogenesis and should facilitate the development of therapeutic strategies that target nonmutated *TP53* in patients with HGSOC.

## Supporting Information

Figure S1 **ST1 in TCGA data.** (Upper panel) Summary of mutations for *TP53* and p53 pathway genes for 15 *TP53* nonmutated patients with HGSOC in TCGA data. *TP53* homozygous deletion is shown in dark blue and heterozygous copy number deletions are shown in light blue in *TP53*_Del track. *MDM2* copy number amplification is shown in red in the *MDM2*_amp track. Mutations in genes that are direct targets of p53 are shown in green in the p53_Target_mut track. (Bottom panel) Hierarchical clustering of TCGA-25-1328 and 33 HGSOC using 45 overlapping genes among the 70 differentially expressed genes.
(PDF)

Figure S2 **Survival analysis.** (Left panel) Overall survival curves for ST1 and ST2. (Right panel) Progression-free survival curves for ST1 and ST2. These survival curves were depicted using the Kaplan-Meier method. p values correspond to the Logrank test comparing the survival curves.
(PDF)

Table S1 **Clinical data.** pT- and FIGO-stages. Two subtypes (ST1 and ST2) are shown in Subtype column.
(XLSX)

Table S2 **Depth and coverage of exome sequencing.** Depth and coverage were calculated using DepthOfCoverage module of GATK.
(XLS)

Table S3 **Depth of coding exons of** ***TP53.*** Depth of ten coding exons of *TP53* (NM_001126112.2) were calculated using SAMtools.
(XLSX)

Table S4 **Somatic** ***TP53*** **mutations.** Functional impacts of missense single nucleotide variants which were evaluated using MutationAssessor 2 are shown in the FIS column.
(XLS)

Table S5 **Copy number deleted regions.** Recurring copy number deleted regions are shown in CNVR (hg18) column. Gene column shows genes which are located in these CNVRs.
(PDF)

Table S6 **Copy number amplified regions.** Recurring copy number amplified regions are shown in CNVR (hg18) column. Gene column shows genes which are located in these CNVRs.
(PDF)

Table S7 **List of p53 direct target genes.** A list of p53 direct target genes were derived from the Pathway Interaction Database (PID).
(XLSX)

Table S8 **CNV segments.** CNV segments were processed using PennCNV. Two subtypes (ST1 and ST2) are shown in Subtype column.
(XLS)

Table S9 **Tumor purity.** Tumor purities were estimated using ASCAT algorithm in the NEXUS copy number software. Two subtypes (ST1 and ST2) which were designated in the current study are shown in Subtype column.
(XLS)

Table S10 **Probes which showed higher expression in ST1.** Forty-four probes (33 genes) are listed. Gene symbols, Agilent probe ID, and genomic positions of the probes were showed in Gene Symbol, Probe ID, and GenomicCoordinates columns, respectively. *N.A.* means not available.
(PDF)

Table S11 **Probes which showed lower expression in ST1.** Forty-five probes (37 genes) are listed. Gene symbols, Agilent probe ID, and genomic positions of the probes were showed in Gene Symbol, Probe ID, and GenomicCoordinates columns, respectively. *N.A.* means not available.
(PDF)

Table S12 **Go groups.** Eighteen GO groups and their component genes are listed.
(XLS)

Table S13 **Statistical tests.** Results of statistical tests (the Kolmogorov-Smirnov test, F test, and *t*-test) for mitosis and DNA helicase genes are shown.
(PDF)

File S1 **Copy number data.** CNVs were called using PennCNV. CNVs are shown in seg.mean column. Numbers of support SNPs are shown in seg.count column.
(TXT)
