# Supplementary material for: Molecular Characterization of an Intact p53 Pathway Subtype in High-Grade Serous Ovarian Cancer
Source: PLoS One. 2014 Dec 2;9(12):e114491. doi: 10.1371/journal.pone.0114491 (PMC4252108; doi:10.1371/journal.pone.0114491)
Supplement: Figure S1 — ST1 in TCGA data. (Upper panel) Summary of mutations for TP53 and p53 pathway genes for 15 TP53 nonmutated patients with HGSOC in TCGA data. TP53 homozygous deletion is shown in dark blue and heterozygous copy number deletions are shown in light blue in TP53_Del track. MDM2 copy number amplification is shown in red in the MDM2_amp track. Mutations in genes that are direct targets of p53 are shown in green in the p53_Target_mut track. (Bottom panel) Hierarchical clustering of TCGA-25-1328 and 33 HGSOC using 45 overlapping genes among the 70 differentially expressed genes. (PDF) [file pone.0114491.s001.pdf]

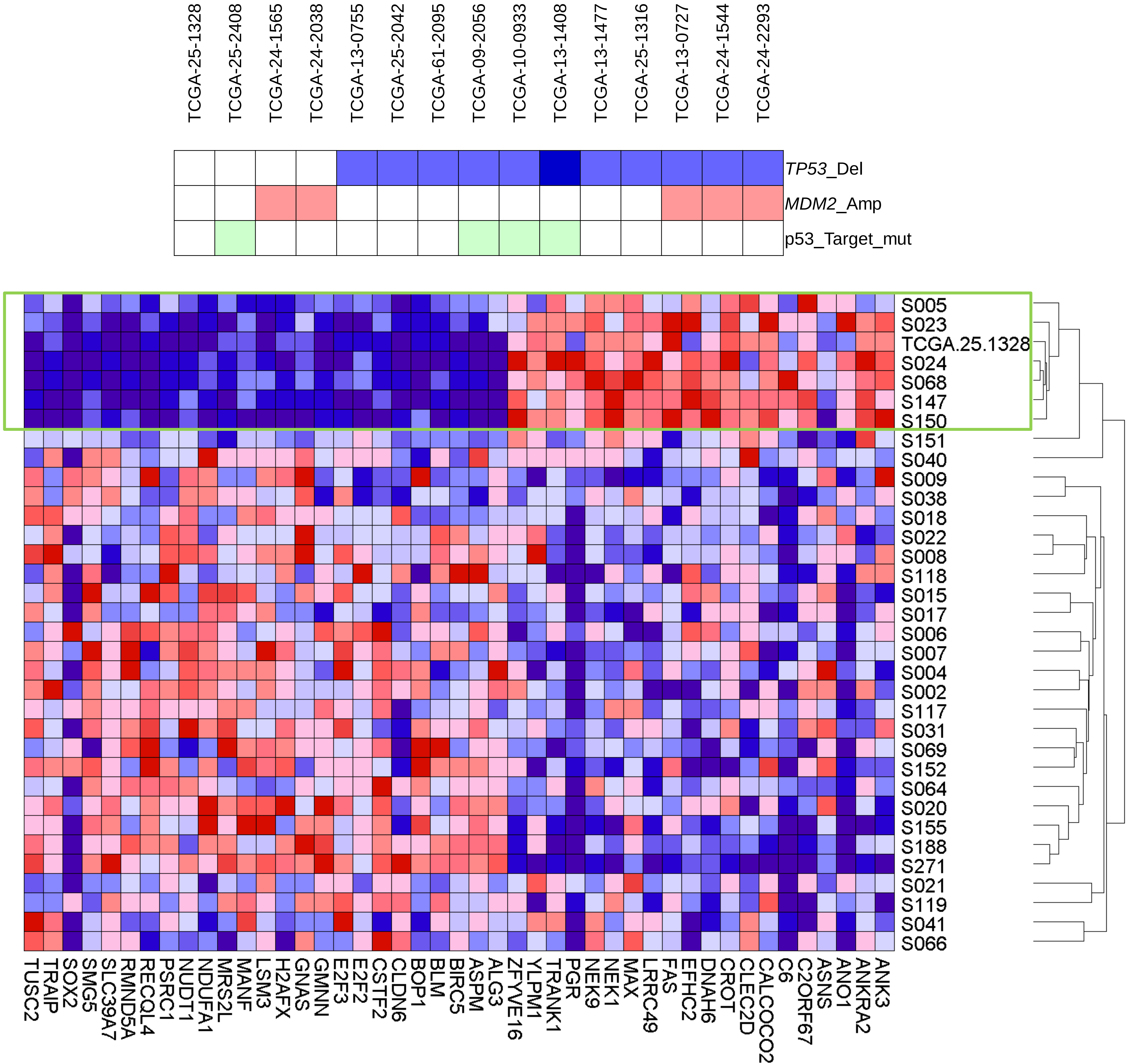
TCGA-25-1328
TCGA-25-2408
TCGA-24-1565
TCGA-24-2038
TCGA-13-0755
TCGA-25-2042
TCGA-61-2095
TCGA-09-2056
TCGA-10-0933
TCGA-13-1408
TCGA-13-1477
TCGA-25-1316
TCGA-13-0727
TCGA-24-1544
TCGA-24-2293
*TP53*_Del
*MDM2*_Amp
p53_Target_mut
S005
S023
TCGA.25.1328
S024
S068
S147
S150
S151
S040
S009
S038
S018
S022
S008
S118
S015
S017
S006
S007
S004
S002
S117
S031
S069
S152
S064
S020
S155
S188
S271
S021
S119
S041
S066
TUSC2
TRAIP
SOX2
SMG5
SLC39A7
RMND5A
RECQL4
PSRC1
NUDT1
NDUFA1
MRS2L
MANF
LSM3
H2AFX
GNAS
GMNN
E2F3
E2F2
CSTF2
CLDN6
BOP1
BLM
BIRC5
ASPM
ALG3
ZFYVE16
YLPM1
TRANK1
PGR
NEK9
NEK1
MAX
LRRC49
FAS
EFHC2
DNAH6
CROT
CLEC2D
CALCOCO2
C6
C2ORF67
ASNS
ANO1
ANKRA2
ANK3
