# Supplementary figures and images for: Molecular Characterization of an Intact p53 Pathway Subtype in High-Grade Serous Ovarian Cancer
Source: PLoS One. 2014 Dec 2;9(12):e114491. doi: 10.1371/journal.pone.0114491 (PMC4252108; doi:10.1371/journal.pone.0114491)

Overall survival

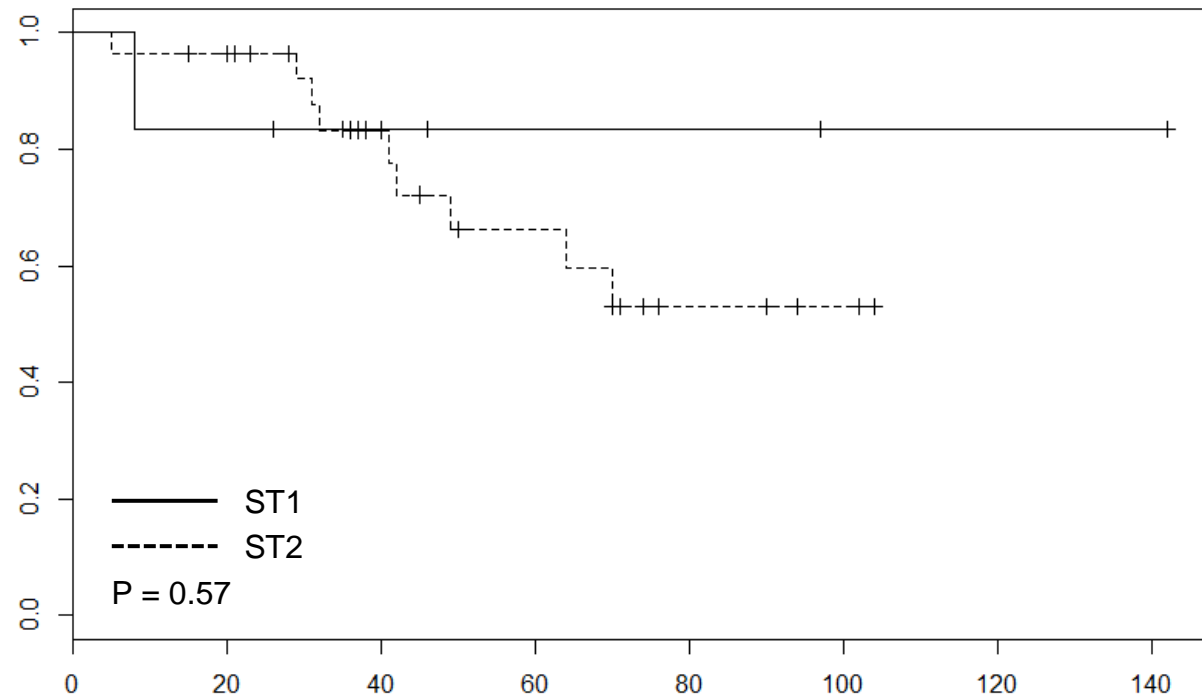

Progression-free survival

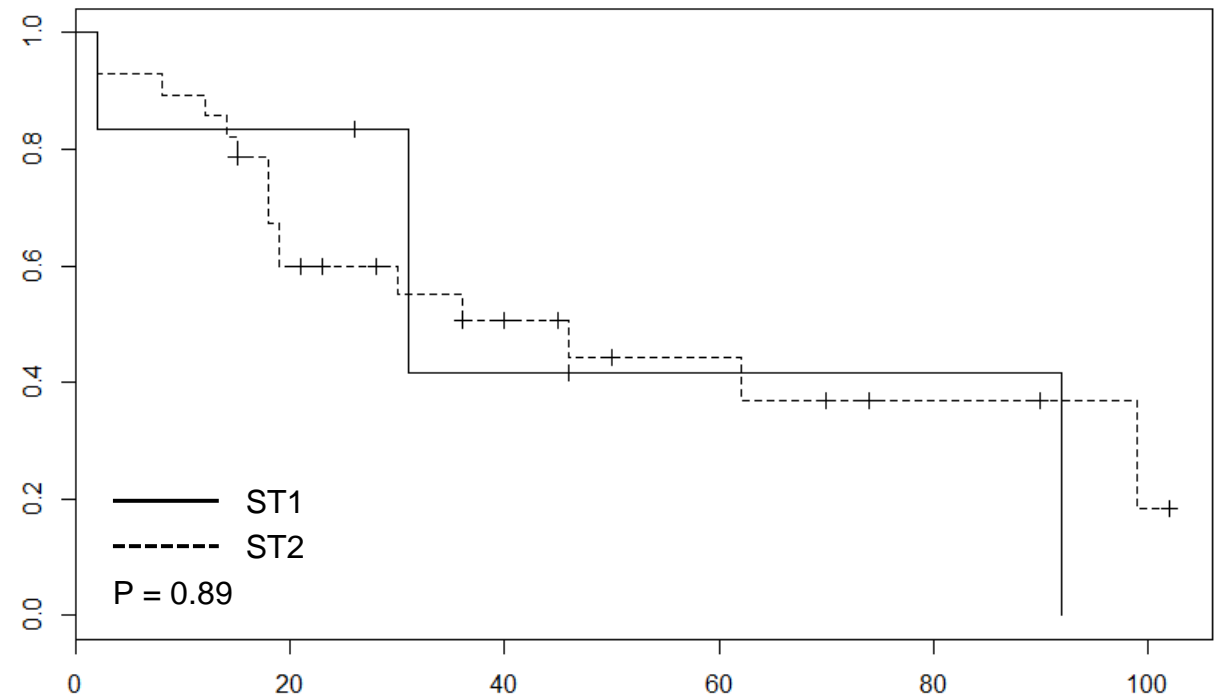

Supplement: Figure S2 — Survival analysis. (Left panel) Overall survival curves for ST1 and ST2. (Right panel) Progression-free survival curves for ST1 and ST2. These survival curves were depicted using the Kaplan-Meier method. p values correspond to the Logrank test comparing the survival curves. (PDF) [file pone.0114491.s002.pdf]
