# Supplementary material for: Molecular Characterization of an Intact p53 Pathway Subtype in High-Grade Serous Ovarian Cancer
Source: PLoS One. 2014 Dec 2;9(12):e114491. doi: 10.1371/journal.pone.0114491 (PMC4252108; doi:10.1371/journal.pone.0114491)
Supplement: Table S5 — Copy number deleted regions. Recurring copy number deleted regions are shown in CNVR (hg18) column. Gene column shows genes which are located in these CNVRs. (PDF) [file pone.0114491.s007.pdf]

CNVR (hg18)	Cytoband	Fisher's exact test	Cases Del	Control Del	Gene
chr4:103571694-103707311	4q24	5.E-08	18	0	*NFKB1*
chr6:130106363-130740491	6q22.33,6q23.1	4.E-07	16	0	*ARHGAP18,C6orf191,L3MBTL3,SAMD3,TMEM200A*
chr17:29925858-30230919	17q12	6.E-07	16	0	*C17orf102,TMEM132E*
chr5:71393645-72053142	5q13.2	6.E-07	16	0	*MAP1B,MRPS27,PTCD2,ZNF366*
chr5:66877750-67197527	5q13.1	6.E-07	17	0	*BC042046*
chr22:34881104-35141765	22q12.3	8.E-07	15	0	*APOL1,APOL2,APOL3,APOL4,APOLIV,AX747758,AX748195,MYH9*
chr18:53265145-53392188	18q21.31	3.E-06	14	0	*FECH,ONECUT2*
chr15:74189059-74665417	15q24.2,15q24.3	2.E-05	12	0	*C15orf27,ETFA,ISL2,KIAA1454,SCAPER*
chr1:25149393-25310537	1p36.11	5.E-05	11	0	*AML2,RUNX3*
