# Supplementary material for: Molecular Characterization of an Intact p53 Pathway Subtype in High-Grade Serous Ovarian Cancer
Source: PLoS One. 2014 Dec 2;9(12):e114491. doi: 10.1371/journal.pone.0114491 (PMC4252108; doi:10.1371/journal.pone.0114491)
Supplement: Table S6 — Copy number amplified regions. Recurring copy number amplified regions are shown in CNVR (hg18) column. Gene column shows genes which are located in these CNVRs. (PDF) [file pone.0114491.s008.pdf]

CNVR (hg18)	Cytoband	Fisher's exact test	Cases Amp	Control Amp	Gene
chr3:186592298-187095547	3q27.2	1.E-07	17	0	*C3orf65,IGF2BP2,LIPH,MAP3K13,SENP2,TMEM41A,UNQ168,p62*
chr6:10615513-10816609	6p24.2	8.E-06	13	0	*C6orf52,GCNT2,IGnT2,PAK1IP1*
chr1:46262506-46947778	1p33,1p34.1	2.E-05	12	0	*AK026430,ATPAF1,C1orf190,C1orf223,DKFZp434A091,DKFZp686E14208,DMBX1,FAAH,KIAA0494,KIAA0807,KNCN,LRRC41,MAST2,MKNK1,MNK1B,MOBKL2C,NSUN4,PIK3R3,POMGNT1,RAD54L,TSPAN1,UDP-GlcNAc,UQCRH*
chr10:21535145-23690109	10p12.2,10p12.31	4.E-04	8	0	*AF10,AF10/CALM,AK055656,ARMC3,BMI1,C10orf114,C10orf140,C10orf67,CALM/AF10,COMMD3,DKFZp434I153,DKFZp779J0967,DNAJC1,MLLT10,MSRB2,PIP4K2A,PTF1A,SPAG6*
