# Supplementary material for: Molecular Characterization of an Intact p53 Pathway Subtype in High-Grade Serous Ovarian Cancer
Source: PLoS One. 2014 Dec 2;9(12):e114491. doi: 10.1371/journal.pone.0114491 (PMC4252108; doi:10.1371/journal.pone.0114491)
Supplement: Table S10 — Probes which showed higher expression in ST1. Forty-four probes (33 genes) are listed. Gene symbols, Agilent probe ID, and genomic positions of the probes were showed in Gene Symbol, Probe ID, and GenomicCoordinates columns, respectively. N.A. means not available. (PDF) [file pone.0114491.s012.pdf]

| Gene Symbol | Probe ID | GenomicCoordinates |
|---|---|---|
| *ANK3* | A_24_P162173 | chr10:61513299-61512486 |
| *ANKRA2* | A_24_P337397 | chr5:72885961-72885067 |
| *ANO1* | A_24_P110831 | chr11:69687269-69687328 |
| *ANO1* | A_24_P87036 | chr11:69712881-69712940 |
| *ASNS* | A_32_P232747 | chr7:97376131-97376072 |
| *C10ORF107* | A_32_P8156 | chr10:63190730-63195724 |
| *C1ORF173* | A_32_P84237 | chr1:74810798-74810739 |
| *C2ORF67* | A_23_P400373 | chr2:210595413-210595354 |
| *C6* | A_23_P92928 | chr5:41178728-41178669 |
| *CALCOCO2* | A_24_P322191 | chr17:44288458-44288517 |
| *CCDC148* | A_23_P411825 | chr2:158903772-158903631 |
| *CCDC17* | A_23_P430800 | chr1:45858487-45858428 |
| *CLEC2D* | A_24_P364335 | chr12:9739644-9739703 |
| *CROT* | A_23_P168669 | chr7:86865934-86865993 |
| *DNAH6* | A_23_P324605 | chr2:84893743-84894642 |
| *DNAH6* | A_24_P231074 | chr2:84866266-84866325 |
| *EFHC2* | A_24_P147927 | chrX:43920531-43908208 |
| *FAS* | A_23_P63896 | chr10:90764525-90764584 |
| *KLHDC1* | A_23_P422766 | chr14:49289150-49289209 |
| *LOC389634* | A_32_P148914 | chr8:12561140-12561081 |
| *LOC389634* | A_32_P221305 | chr12:8400913-8400854 |
| *LOC442006* | A_24_P33429 | chr2:4539711-4539669 |
| *LRRC49* | A_23_P129174 | chr15:69129271-69129330 |
| *MAX* | A_23_P205549 | chr14:64611815-64611756 |
| *N.A.* | A_24_P410117 | chr2:204345675-204345616 |
| *N.A.* | A_24_P938006 | chr14:073594870-073594785 |
| *N.A.* | A_32_P120484 | chr4:159850880-159850939 |
| *N.A.* | A_32_P157465 | chr3:115534333-115534274 |
| *N.A.* | A_32_P204239 | chr7:105463809-105463868 |
| *N.A.* | A_32_P211765 | chr12:8400853-8400826 |
| *NEK1* | A_23_P124427 | chr4:170551986-170551927 |
| *NEK9* | A_23_P3131 | chr14:74620866-74620807 |
| *PGR* | A_23_P138938 | chr11:100417978-100417919 |
| *PGR* | A_32_P49199 | chr11:100406127-100406068 |
| *POLK* | A_24_P303160 | chr5:74928485-74928544 |
| *SLC23A1* | A_23_P21990 | chr5:138735644-138735585 |
| *SPATA18* | A_23_P407112 | chr4:52657932-52657991 |
| *SPATA18* | A_32_P216841 | chr4:52645858-52645917 |
| *TRANK1* | A_23_P91859 | chr3:36844548-36844489 |
| *TTC8* | A_32_P169735 | chr14:88413494-88413553 |
| *VWA3A* | A_23_P390665 | chr16:22071447-22074438 |
| *WDR49* | A_23_P356154 | chr3:168679441-168679382 |
| *YLPM1* | A_23_P3128 | chr14:74371740-74371799 |
| *ZFYVE16* | A_24_P8349 | chr5:79782137-79783094 |
