# Supplementary material for: Molecular Characterization of an Intact p53 Pathway Subtype in High-Grade Serous Ovarian Cancer
Source: PLoS One. 2014 Dec 2;9(12):e114491. doi: 10.1371/journal.pone.0114491 (PMC4252108; doi:10.1371/journal.pone.0114491)
Supplement: Table S11 — Probes which showed lower expression in ST1. Forty-five probes (37 genes) are listed. Gene symbols, Agilent probe ID, and genomic positions of the probes were showed in Gene Symbol, Probe ID, and GenomicCoordinates columns, respectively. N.A. means not available. (PDF) [file pone.0114491.s013.pdf]

Gene Symbol	Probe ID	GenomicCoordinates
ALG3	A_24_P414269	chr3:185442980-185442921
APLN	A_24_P404822	chrX:128607551-128607492
ASPM	A_23_P52017	chr1:195320136-195320077
ASPM	A_24_P911179	chr1:195379806-195379747
BIRC5	A_32_P188921	chr17:73731940-73731881
BLM	A_23_P88630	chr15:89155804-89155863
BOP1	A_23_P43800	chr8:145457384-145457241
C15ORF42	A_32_P109296	chr15:87972140-87972199
CDCA2	A_23_P385861	chr8:25421173-25421232
CLDN6	A_23_P37942	chr16:3005315-3005256
CSTF2	A_23_P148372	chrX:99979062-99979910
E2F2	A_23_P408955	chr1:23705579-23705520
E2F3	A_23_P385034	chr6:20601824-20601883
FANCD2	A_23_P143994	chr3:10118006-10118065
GMNN	A_23_P19712	chr6:24894087-24894146
GNAS	A_24_P273666	chr20:56919399-56919458
H2AFX	A_24_P38895	chr11:118469860-118469801
KANK4	A_32_P51237	chr1:62475913-62475854
LOC440944	A_24_P479645	chr3:9407197-9407138
LSM3	A_23_P132863	chr3:14214790-14214849
LSM3	A_23_P421563	chr3:14214691-14214750
LSM3	A_24_P29001	chr3:14214599-14214658
MANF	A_23_P132793	chr3:51401727-51401786
MRS2	A_23_P111373	chr6:24532216-24532275
N.A.	A_24_P922497	chr8:41521751-41521810
N.A.	A_32_P165990	chr5:332249-332308
N.A.	A_32_P68055	chr8:95721499-95721440
N.A.	A_32_P91519	chr2:219493522-219493581
NDUFA1	A_24_P348649	chrX:118891323-118891382
NUDT1	A_23_P134295	chr7:2257086-2257145
PIF1	A_23_P323749	chr15:62903111-62903052
PIF1	A_23_P416468	chr15:62895028-62894969
POC1A	A_23_P212284	chr3:52084553-52084494
PSRC1	A_23_P46539	chr1:109624041-109623982
RECQL4	A_23_P71558	chr8:145707730-145707671
RMND5A	A_23_P257753	chr2:86854652-86854711
SKA3	A_23_P340909	chr13:20626190-20626131
SLC39A7	A_23_P70571	chr6:33279778-33279837
SMG5	A_23_P322043	chr1:154485978-154485919
SOX2	A_23_P401055	chr3:182914571-182914630
TCF19	A_24_P349965	chr6:31239778-31239837
TRAIP	A_23_P170491	chr3:49841148-49841089
TUSC2	A_23_P251562	chr3:50337451-50337392
UBE2T	A_23_P115482	chr1:200567588-200567529
YWHAG	A_23_P123022	chr7:75797367-75797308
