# Supplementary material for: Molecular Characterization of an Intact p53 Pathway Subtype in High-Grade Serous Ovarian Cancer
Source: PLoS One. 2014 Dec 2;9(12):e114491. doi: 10.1371/journal.pone.0114491 (PMC4252108; doi:10.1371/journal.pone.0114491)
Supplement: Table S13 — Statistical tests. Results of statistical tests (the Kolmogorov-Smirnov test, F test, and t-test) for mitosis and DNA helicase genes are shown. (PDF) [file pone.0114491.s015.pdf]

Gene	Probe	Kolmogorov-Smirnov test (ST1)	Kolmogorov-Smirnov test (ST2)	F test	t-test (var.equal=T)	t-test (var.equal=F)
NEK1	A_23_P124427	0.87	0.55	0.19	0.0000019	-
NEK9	A_23_P3131	0.78	0.82	0.037	-	0.010
ASPM	A_23_P52017	0.84	0.67	0.020	-	0.0000015
ASPM	A_24_P911179	0.68	0.55	0.0017	-	0.00000019
BIRC5	A_32_P188921	0.87	0.17	0.0043	-	0.0000098
CDCA2	A_23_P385861	0.67	0.35	0.070	0.0035	-
SKA3	A_23_P340909	0.53	0.74	0.073	0.000078	-
Gene	Probe	Kolmogorov-Smirnov test (ST1)	Kolmogorov-Smirnov test (ST2)	F test	t-test (var.equal=T)	t-test (var.equal=F)
BLM	A_23_P88630	0.99	0.56	0.0027	-	0.0000037
PIF1	A_23_P323749	0.72	0.20	0.011	-	0.000017
PIF1	A_23_P416468	0.29	0.29	0.023	-	0.0000045
RECQL4	A_23_P71558	0.69	0.32	0.0020	-	0.00000043
